# Targeting Mitochondria by Zn(II)*N*-Alkylpyridylporphyrins: The Impact of Compound Sub-Mitochondrial Partition on Cell Respiration and Overall Photodynamic Efficacy

**DOI:** 10.1371/journal.pone.0108238

**Published:** 2014-09-24

**Authors:** Ahmad M. Odeh, James D. Craik, Rima Ezzeddine, Artak Tovmasyan, Ines Batinic-Haberle, Ludmil T. Benov

**Affiliations:** 1 Department of Biochemistry, Faculty of Medicine, Kuwait University, Kuwait City, Kuwait; 2 Department of Radiation Oncology, Duke University Medical Center, Durham, North Carolina, United States of America; Auburn University, United States of America

## Abstract

Mitochondria play a key role in aerobic ATP production and redox control. They harness crucial metabolic pathways and control cell death mechanisms, properties that make these organelles essential for survival of most eukaryotic cells. Cancer cells have altered cell death pathways and typically show a shift towards anaerobic glycolysis for energy production, factors which point to mitochondria as potential culprits in cancer development. Targeting mitochondria is an attractive approach to tumor control, but design of pharmaceutical agents based on rational approaches is still not well established. The aim of this study was to investigate which structural features of specially designed Zn(II)*N*-alkylpyridylporphyrins would direct them to mitochondria and to particular mitochondrial targets. Since Zn(II)*N*-alkylpyridylporphyrins can act as highly efficient photosensitizers, their localization can be confirmed by photodamage to particular mitochondrial components. Using cultured LS174T adenocarcinoma cells, we found that subcellular distribution of Zn-porphyrins is directed by the nature of the substituents attached to the *meso* pyridyl nitrogens at the porphyrin ring. Increasing the length of the aliphatic chain from one carbon (methyl) to six carbons (hexyl) increased mitochondrial uptake of the compounds. Such modifications also affected sub-mitochondrial distribution of the Zn-porphyrins. The amphiphilic hexyl derivative (ZnTnHex-2-PyP) localized in the vicinity of cytochrome c oxidase complex, causing its inactivation during illumination. Photoinactivation of critical cellular targets explains the superior efficiency of the hexyl derivative in causing mitochondrial photodamage, and suppressing cellular respiration and survival. Design of potent photosensitizers and redox-active scavengers of free radicals should take into consideration not only selective organelle uptake and localization, but also selective targeting of critical macromolecular structures.

## Introduction

The role of mitochondria in aerobic energy production, redox control, and various unique metabolic pathways, makes these organelles essential for survival of most eukaryotic cells. Mitochondria play a key role in cell death mechanisms, and cancer development is associated with suppression of apoptotic pathways [1]. Although tumor cell metabolism is characterized by a shift towards increased importance of glycolysis [2], mitochondria remain important for cell survival. Given their metabolic importance and special characteristics (particularly the transmembrane electrochemical potential of the mitochondrial inner membrane), developing mitochondria-directed chemotherapy through targeting mitochondrial structures and functions is becoming an attractive approach to tumor cell control [3], [4].

Photosensitizers are compounds capable of absorbing light energy and by transferring it to other, non-absorbing molecules, are able to induce chemical reactions. The photodynamic reactions start with absorption of a photon by the photosensitizer raising it to an excited state. Reacting with oxygen, the excited photosensitizer molecule can generate either singlet oxygen (^1^O_2_) or superoxide anion radical (O_2_^.−^) [5]. For reasons discussed in detail elsewhere [6], singlet oxygen is considered the main cause of phototoxicity in photodynamic therapy. It is a powerful oxidant that can react with a multiplicity of biomolecules, including lipids, proteins, and nucleic acids [7]. The rate constants for the reaction of ^1^O_2_ with different biomolecules vary over a wide range, which results in selective damage to particular molecules and structures [8]. Singlet oxygen lifetime in cells is less than 4.0 µs, limiting its diffusion distance to a maximum of 150 nm [9], which means that the initial damage caused by singlet oxygen produced at a particular intracellular location will be limited in space. Therefore, cell damage and its consequences, including induction and execution of cell death pathways, will depend on the location of the photosensitizer [10], [11]. Consequently, photodynamic therapy efficacy is dependent not only on the selective accumulation but also on the subcellular localization of a photosensitizer. Photosensitizers that localize to mitochondria are more efficient in killing cells than those that localize at other cellular sites [12]. Rational design of mitochondria-targeting agents requires detailed understanding of molecular features that guide and direct the molecule to particular structures. In general, two principles have been used to give a molecule mitochondria-targeting ability: (1) Attachment of a mitochondria-targeting peptide sequence; and (2) Combination of lipophilic residues with cationic groups, thus exploiting the high membrane potential across the inner mitochondrial membrane [13], [14].

Using porphyrin as a basic structure, in the current study we investigated molecular modifications that direct the compound to mitochondria. Our results demonstrate that attachment of positively charged pyridyl nitrogens at *meso* positions and six-carbon aliphatic chains at the porphyrin ring periphery, directs the molecule to mitochondria. The Zn(II)*meso*-tetrakis(*N*-hexylpyridinium-2-yl)porphyrin designed in this manner localizes to mitochondria and efficiently photoinactivates cytochrome c oxidase. In addition to hampering energy production, photoinactivation of cytochrome c oxidase has been shown to cause secondary generation of superoxide by the electron transport chain; this may act together with primary photodynamic damage to drive tumor cell killing [4].

## Materials and Methods

### Zn porphyrins

Synthesis and characterization of Zn porphyrins was performed as previously described [15]. Lipophilicity was studied by determining partition between octanol and water and was expressed as partition coefficient, log P_ow_. In parallel, lipophilicity was assessed chromatographically [16] and was expressed as chromatographic retention factor, R*_f_*.

### Cell culture

Experiments were performed using human colon adenocarcinoma cell line LS174T [17]. The cell line was maintained in culture as previously described [18]. Monolayer cultures were grown in RPMI 1640 medium supplemented with 10% fetal bovine serum, 1% L-glutamine, 1% penicillin/streptomycin as an antibacterial agent and 0.1% amphotericin as an antifungal agent. Cultures were maintained at 37°C and 5% CO_2_ and used for experiments at 80–90% confluency. Illumination was performed in PBS as previously described [15]. Two 5 W white fluorescent light tubes fixed in a light box with a translucent screen (SARTORIUS-GMBH, Göttingen) provided a fluence of 2.0 mW/cm^2^.

In all experiments, controls that were illuminated but not containing Zn porphyrins, and controls containing Zn porphyrins but not illuminated, were processed in parallel.

### MTT reduction assay

The 3-(4,5-dimethyl-thiazol-2-yl)-2,5-diphenyl-tetrazolium bromide (MTT) test is a surrogate assay for cell viability based on enzymatic reduction of the water-soluble yellow MTT dye to purple formazan. The concentration of the formazan product is proportional to the number of metabolically active cells in the culture [19].

To determine the effect of phototreatment on LS174T cell viability, cells were seeded in flat-bottomed 96-well micro-culture plates at a density of 5×10^4^ cells per well and the volume in each well was adjusted to 100 µl with fresh medium. Cells were incubated overnight to attach. Sterile solutions of photosensitizers were added to each well and incubated for 24 hours in the dark. The cells were then washed with PBS and were illuminated for 30 minutes in PBS. After illumination PBS was replaced with fresh medium and 10 µl of 5 mg/ml MTT dissolved in PBS was directly added to each well. After 3 hours of incubation at 37°C, 100 µl of 10% SDS in 0.01M HCl were added to each well and kept overnight to stop the reaction and to solubilize formazan crystals. Absorbance was measured at 560 nm and 650 nm (background) using a micro-plate reader. Samples were run in quadruplicate. Controls containing all the components except photosensitizers, negative controls not containing cells, and dark controls containing all the components and the highest concentrations of photosensitizers without being illuminated were run in parallel.

### Subcellular localization of Zn porphyrins

Localization of the studied Zn porphyrins was investigated by confocal fluorescence microscopy as described previously [15]. In brief, adherent cells were incubated with Zn porphyrins in the dark, co-stained with MitoTracker Green FM (200 nM), DIOC6(3) iodide (1.5 µM) or Lysosensor Green DND-189 (3 µM), fixed and washed; images were obtained using a Zeiss LSM META confocal fluorescence microscope fitted with Zeiss AxioCam HRc camera.

### Isolation of mitochondria

#### Mitochondrial preparation from rat liver

The Animal Ethical Committee (Health Sciences Center, Kuwait University) guidelines were followed for handling and treatment of animals. Rats were anesthetized with pentobarbital sodium (40 mg/kg, intraperitoneally) and were sacrificed by exsanguination. Rat liver was rapidly excised and perfused with ice-cold homogenization buffer consisting of 0.25 M sucrose, 5 mM HEPES, and 1 mM EDTA, pH 7.2. All the steps in the isolation protocol were performed on ice with chilled media. The liver was weighed and sucrose buffer was added at a volume 4 times that of the liver weight (1∶4 ratio). The liver was minced into small fragments and homogenized in sucrose buffer by nine complete strokes using a Dounce homogenizer fitted with a tight pestle. The suspension was centrifuged at 1000×g for 10 minutes at 4°C. The supernatant was centrifuged again for 5 more minutes under the same conditions. The supernatant was then centrifuged at 8000×g for 10 minutes at 4°C. The pellet was resuspended in 0.5 ml isotonic sucrose buffer and used for experiments directly after preparation. The protein concentration in the mitochondrial suspension was determined by the Lowry assay [20].

#### Isolation of mitochondria from LS174T cells

A standard protocol including the use of digitonin in the homogenization buffer was applied since inclusion of digitonin markedly shortens the isolation procedure. LS174T cells were grown to 80–90% confluence and were scraped and resuspended in 1 ml PBS; this cell suspension was centrifuged at 14,000×g for 20 seconds using an Eppendorf 5414 centrifuge. The pellet was resuspended quickly in 1 ml buffer (0.25 M sucrose, 3 mM imidazole, pH 7.4) containing 1 mg/ml digitonin, followed by immediate centrifugation at the same speed for another 20 seconds [21].

### Respiration

The effect of different Zn porphyrins on respiratory activity of LS174T cells and mitochondrial suspensions was assessed using an oxygen monitoring system equipped with a Clark-type oxygen electrode (YSI model 5300 Biological Oxygen Monitor). Cells were loaded with photosensitizers and illuminated as described above, except that 6-well plates were used instead of 96-well plates. The cells were then scraped in the dark and cell suspension from each well (adjusted to 4 ×10^6^ cells per ml) was added to 3 ml PBS containing 2% glucose in the oxygraph chamber to measure respiration.

Respiration of isolated intact mitochondria was measured after 15 minutes of dark incubation with photosensitizers followed by illumination. The oxygen electrode was equilibrated to obtain a baseline with a solution of PBS, 5 mM succinate and 50 µM ADP.

### Cytochrome c oxidase assay

Bovine heart cytochrome *c* was dissolved in 50 mM phosphate buffer, pH 7.5 to give a final concentration of 1.2 mM. A pinch of Na_2_S_2_O_4_ was used to reduce the solution of cytochrome *c*. After reduction, the solution was subjected to air bubbling for 15–20 minutes in order to oxidize excess dithionite. Reduced cytochrome c was used immediately after reduction or stored overnight in the freezer in the dark [22].

Cytochrome *c* oxidase was assayed in intact cells and in intact and permeabilized isolated mitochondria. Mitochondria were permeabilized by sonication (Soniprep 150, MSE, UK, sonicator) for three cycles (each cycle ran for 10 seconds, with 8 seconds resting time between cycles) then diluted in phosphate buffer to give a protein concentration of 18 mg/ml. Mitochondrial suspensions were preincubated with photosensitizers in the dark for 30 minutes and were then illuminated for 30 minutes. Dark controls and controls incubated and illuminated without PS were run in parallel.

For assay of cytochrome c oxidase activity, the reaction mixture contained 60 µM cytochrome *c* and the sample (10 µl of phototreated or control mitochondria) in 50 mM phosphate buffer, pH 7.5 to a final volume of 1 ml [22]. The reaction rate was measured by recording the change of absorbance at 550 nm for 60 seconds.

### Glutamate dehydrogenase assay

The catalytic activity of glutamate dehydrogenase was assayed by measuring decrease in absorbance at 340 nm [23]. The reaction mixture contained 100 µM NAD(P)H, 100 µl of phototreated mitochondria and 5 mM α-ketoglutarate in 0.1 M potassium phosphate buffer. Assays were also conducted using mitochondria that were first subjected to disruption by sonication, followed by phototreatment prior to enzyme assay.

### Statistical analysis

Each experiment was repeated at least three times. The data were analyzed using the student t-test; a value p≤ 0.05 was taken to indicate significance, which is indicated with a star (*).

## Results

### Effect of structural modifications on lipophilicity

**Figure 1:**
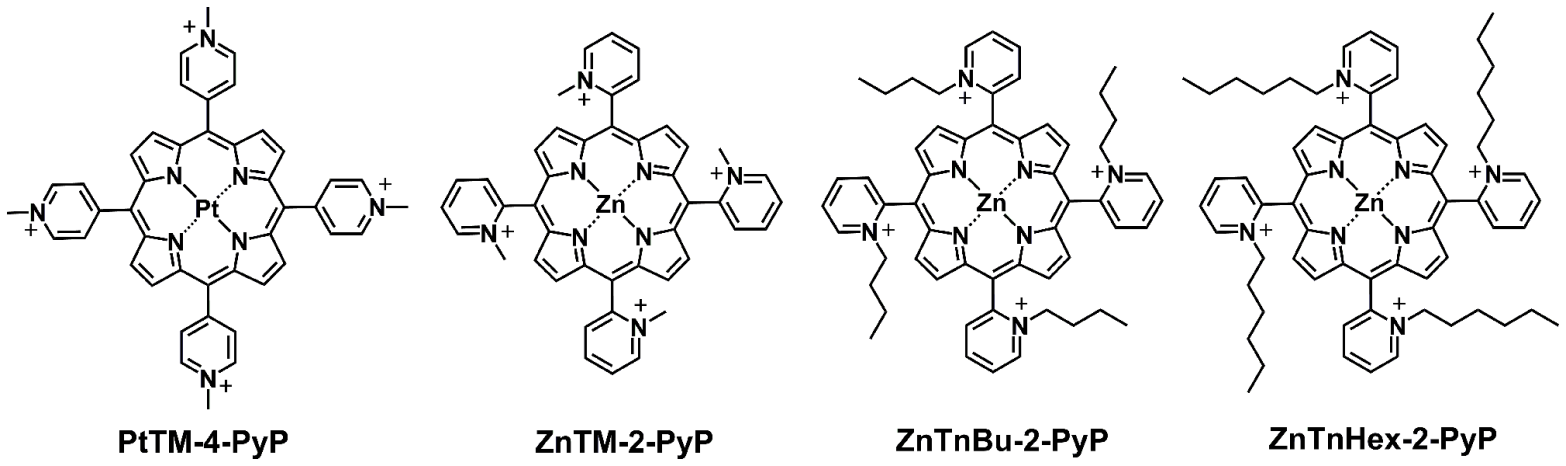
Structures of *ortho* Zn(II) *meso*-tetrakis(*N*-alkylpyridinium-2-yl)porphyrins investigated in this study and the *para* platinum analog (PtTM-4-PyP) [36] of the methyl Zn-porphyrin (ZnTM-2-PyP). PtTM-4-PyP, Pt(II) *meso*-tetrakis(*N*-methylpyridinium-4-yl)porphyrin; Zn porphyrin, Zn(II) *meso*-tetrakis(*N*-alkylpyridinium-2-yl)porphyrin, alkyl being methyl (M, ZnTM-2-PyP), n-butyl (nBu, ZnTnBu-2-PyP), n-hexyl (nHex, ZnTnHex-2-PyP).

In previous investigations, cationic hydrophilic methyl isomers (*ortho* isomer, ZnTM-2-PyP is shown in **Fig. 1**), demonstrated superior photodynamic activity against Gram-negative bacteria and cancer cells than the porphyrin-based photosensitizer currently approved for clinical use, Hematoporphyrin D [24]–[27]. In order to improve its cellular uptake, and consequently its photoefficiency, the lipophilicity of the *ortho* isomer was increased by increasing the length of the aliphatic chain linked to the *meso* pyridyl nitrogen. Both, P*_ow_* and R*_f_* assays (**Table 1**) demonstrated that lipophilicity of the Zn porphyrin is increased by approximately one order of magnitude for each CH_2_ moiety introduced in the aliphatic side chain. This allows gradual increase of the lipophilicity of the molecule by keeping the core structure and the positive charges unchanged. Our previous studies demonstrated that increasing the lipophilicity of the Zn porphyrin improved its cellular uptake and photodynamic efficacy [15].

**Table 1:** Lipophilicity of Zn(II) *meso*-tetrakis(*N*-alkylpyridinium-2-yl)porphyrins.

Zn porphyrin	nC	Log *P*_ow_	*R*_f_
ZnTM-2-PyP^4+^	1	−9.93 (est.)	0.129
ZnTE-2-PyP^4+^	2	−8.71 (est.)	0.209
ZnTnBu-2-PyP^4+^	4	−6.38 (det.)	0.374
ZnTnHex-2-PyP^4+^	6	−3.65 (det.)	0.504

Lipophilicity was determined from the partition coefficient between n-octanol and water (log *P*_ow_) and chromatographic retention factor (*R*_f_, compound path/solvent path, determined on silica gel plates using 1∶1:8 KNO_3(sat)_:H_2_O:acetonitrile as a mobile phase).

### Photoinduced suppression of metabolic activity and induction of mitochondrial damage

**Figure 2:**
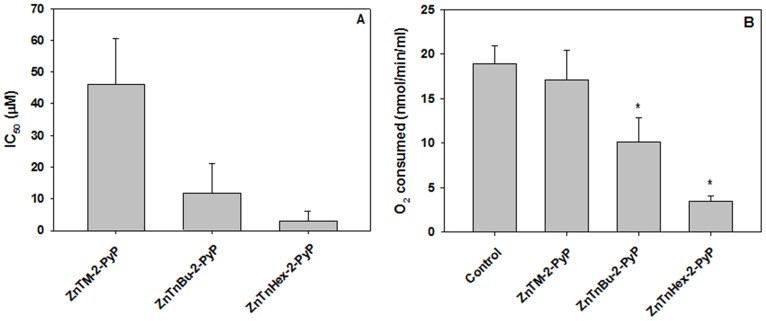
Effect of phototreatment with Zn porphyrins on MTT reduction and respiration. Cells were preincubated for 24 h with Zn porphyrins, washed, resuspended in PBS and illuminated for 30 minutes. A: MTT reduction by cells illuminated after preincubation with *ortho* methyl, butyl or hexyl Zn porphyrins. Concentrations of compounds causing 50% inhibition of MTT reduction (IC_50_) compared to controls are presented; B: Oxygen consumption by cells preincubated with 5 µM Zn porphyrins and illuminated. Mean ± S.E. is presented (n = 4); * p<0.05 compared to control.

Elongation of the alkyl chain attached to the pyridyl nitrogen at the *meso* position increased the photodynamic activity of the Zn porphyrin. **Fig. 2** **A** shows that under the conditions of the experiment, the methyl analog displayed the lowest activity in suppressing MTT reduction, and that elongation of the chain to 6 carbons resulted in dramatic gain of photodynamic efficiency. Among the reasons for higher efficiency is improved cellular uptake [5]. No effect on MTT reduction was observed if cells loaded with Zn Porphyrins were not illuminated. This finding is in agreement with a study that showed no dark toxicity of *para* Zn-porphyrin analogs at concentrations up to 30 µM [28].

When aerobic respiration was determined after illumination, a pattern similar to the suppression of MTT reduction was observed **(** **Fig. 2** **B)**. No suppression of respiration occurred if cells preincubated with Zn porphyrins were kept in the dark or if cells were exposed to light in the absence of Zn porphyrin.

**Figure 3:**
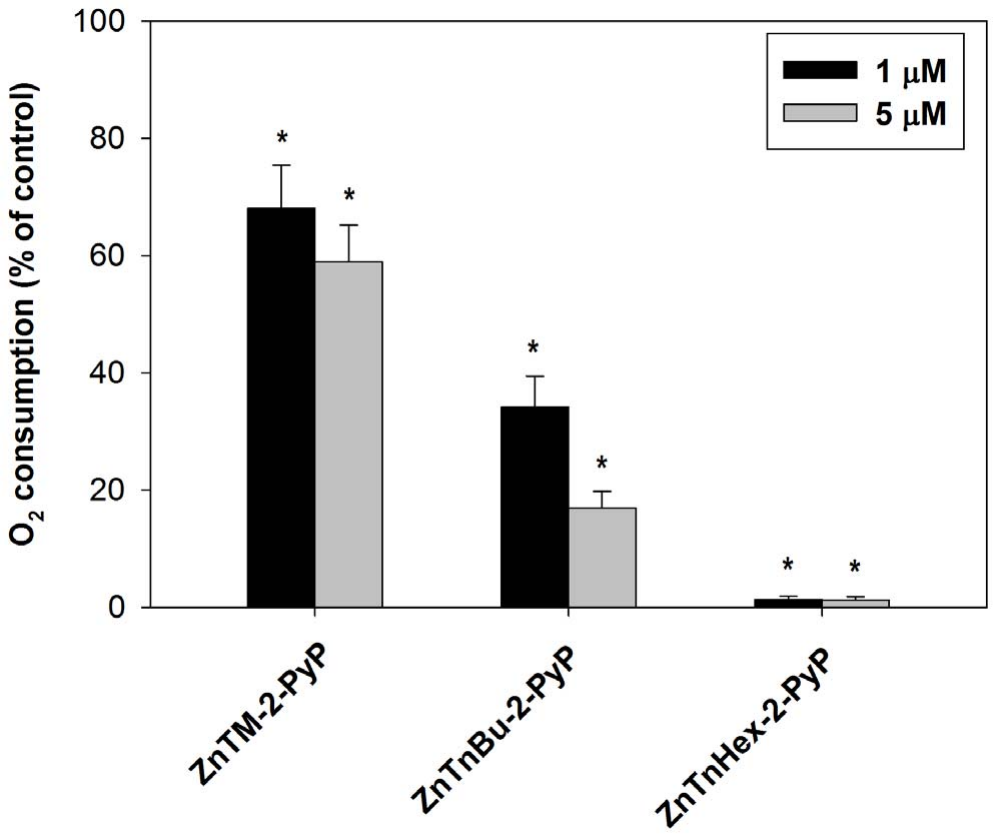
Effect of phototreatment on respiration by isolated mitochondria. Suspensions of intact mitochondria were incubated for 15 minutes with 1 and 5 µM ZnTM-2-PyP, ZnTnBu-2-PyP or ZnTnHex-2-PyP and were then illuminated for 30 minutes. Dark controls and controls with mitochondria illuminated in the absence of photosensitizer were run in parallel. Respiration rate of untreated mitochondria was taken as 100%. (n = 3); * p<0.05 compared to control.

Oxygen consumption by cells is mainly dependent on mitochondrial respiration; therefore it may be assumed that suppression of O_2_ consumption reflects mitochondrial damage. When isolated mitochondria were preincubated with Zn porphyrins and illuminated, suppression of respiration was readily observed; approximately 10 fold lower concentration of ZnTnHex-2-PyP was required for isolated mitochondria than for intact cells to achieve complete suppression of respiration (**Fig. 3**). This result suggests that in intact cells, accumulation of ZnTnHex-2-PyP to sufficiently high concentrations in mitochondria is obstructed by various barriers, and/or that the photosensitizer is diluted by binding to other cellular structures or partitioning into other compartments. A comparison between ZnTM-2-PyP and ZnTnHex-2-PyP shows (**Fig. 3**) that in isolated mitochondria 1 µM concentration of the amphiphilic hexyl photosensitizer was enough to completely suppress respiration, while the effect of the hydrophilic methyl analog, even at 5 µM, barely reached 40%. No effect of Zn porphyrins on respiration was observed when mitochondrial suspensions were kept in dark or when samples were photoirradiated in the absence of Zn porphyrins.

### Subcellular localization

**Figure 4:**
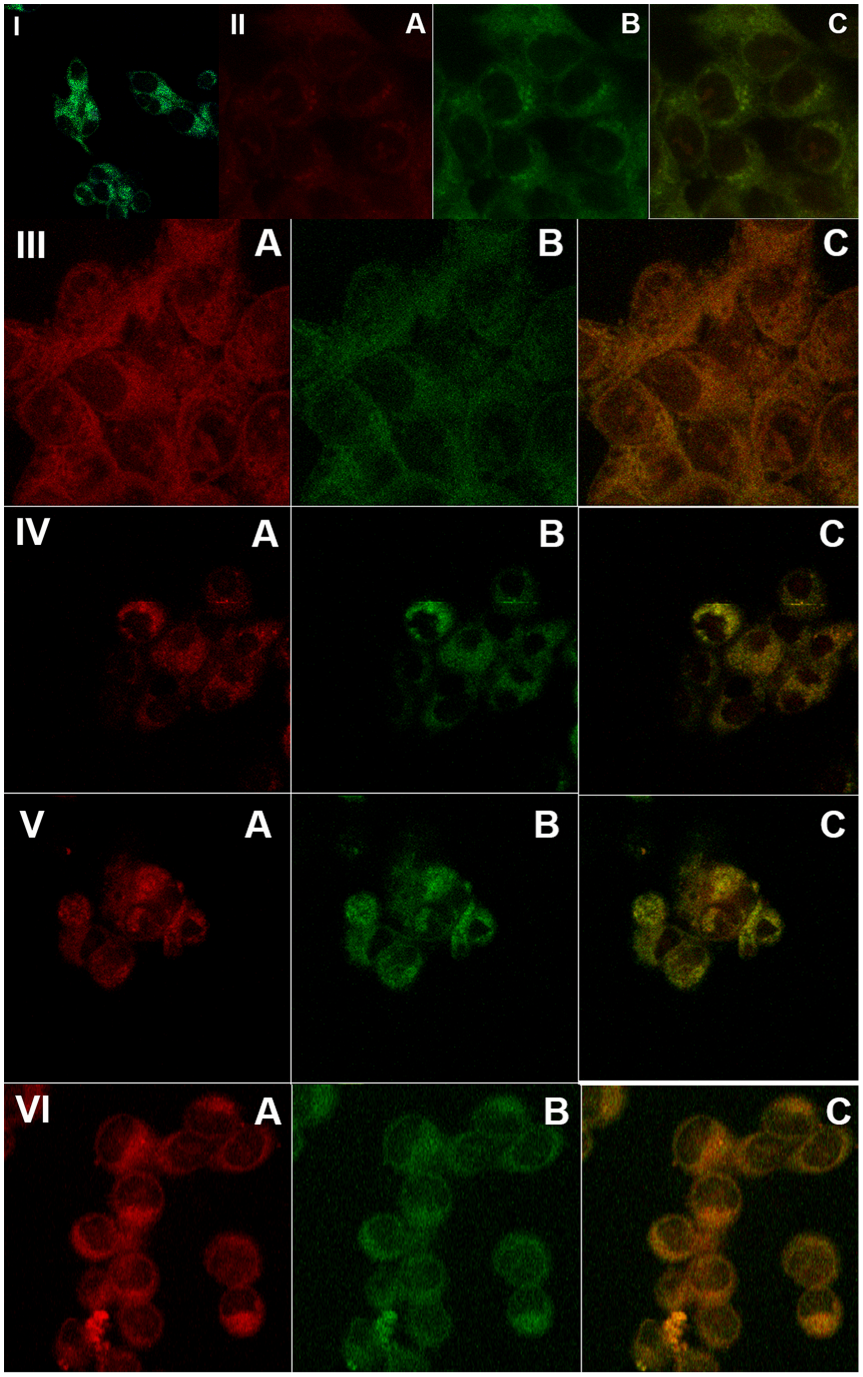
Fluorescence microscopy images of cells preincubated with Zn-porphyrins. I: ZnTM-2-PyP; II: ZnTE-2-PyP; III, IV: ZnTnBu-2-PyP; V, VI: ZnTnHex-2-PyP. A: Cells incubated for in the dark with Zn-porphyrin; B: Costaining with DIOC6(3) iodide (II, IV, V), Lysosensor (III), or MitoTracker (VI) C: Overlay.

Since PSs can relocate upon illumination, mitochondrial damage might not reflect the initial distribution of the PSs. To investigate such a possibility, localization of Zn porphyrins was determined by co-staining with organelle-specific fluorescent markers and fluorescence microscopy. Fluorescent images of cells preincubated with the hydrophilic ZnTM-2-PyP show that the porphyrin dispersed in the cytosol and remained outside the nucleus (Fig. 4, I). As mentioned before [15], even though no bleaching of ZnTM-2-PyP was observed during illumination of the cells, under the intense light of the fluorescent microscope ZnTM-2-PyP bleached very rapidly, which did not allow acquisition of good quality colocalization images. Instead, photomicrographs of its ethyl analog, ZnTE-2-PyP, which demonstrated similar subcellular distribution [15], were presented. Our previous investigations revealed that the hydrophilic Zn-porphyrins localized predominantly in the lysosomes [15]. Based on the presence of yellowish color arising from the overlay of the red (Zn-porphyrin) and the green (fluorescent organelle marker), it can be concluded that very little of the hydrophilic Zn-porphyrin is taken by the endoplasmic reticulum or mitochondria (Fig. 4, II; image with DIOC6(3) iodide shown, MitoTracker similar). Increasing the length of the carbon chain from one/two to four carbon atoms, increased binding of the Zn-porphyrin to the endoplasmic reticulum and mitochondria. Fig. 4, III shows that substantial fraction of ZnTnBu-2-PyP can be found in lysosomes, but compared to ZnTE-2-PyP, much more is dispersed in the endoplasmic reticulum and mitochondria (Fig. 4, IV). Further elongation of the aliphatic chain to six carbon atoms decreased distribution to the endoplasmic reticulum (Fig. 4, V), but strongly increased the uptake of the Zn-porphyrin by the mitochondria (Fig. 4, VI). No partition of the hexyl derivative to lysosomes was detected. Using *para* methyl and octyl *meso*-substituted analogs, Pavani and collaborators found that insertion of Zn into the porphyrin ring decreased partition of photosensitizers to mitochondria [29]. It is important to stress, however, that *ortho* and *para* isomers of the same Zn porphyrin can display quite different subcellular distributions [15]. In support of our previous report [15], in the range 5–20 µM localization of Zn-porphyrins was concentration-independent.

### Photoinactivation of cytochrome c oxidase

**Figure 5:**
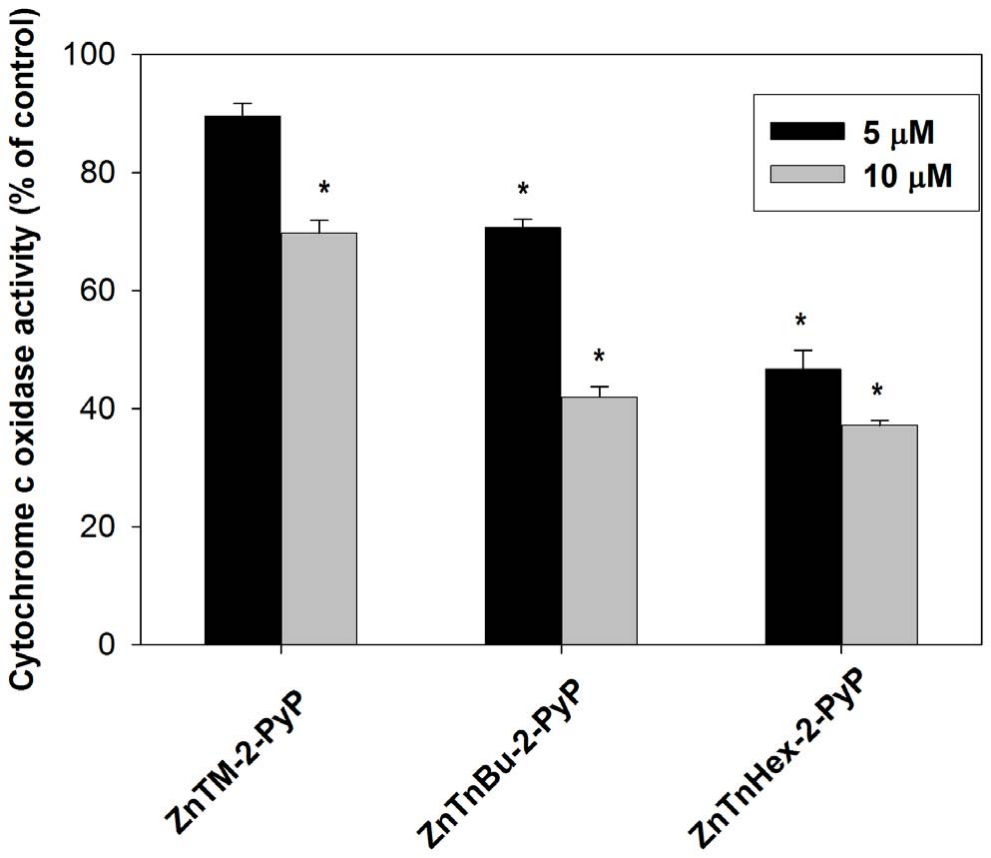
Photoinactivation of cytochrome c oxidase in intact cells. Cells were washed after 24 hours incubation with 5 and 10 µM of ZnTM-2-PyP, ZnTnBu-2-PyP or ZnTnHex-2-PyP and were illuminated for 30 minutes. Cytochrome c oxidase activity was determined in cell-free lysates. Data are expressed as mean ± SE (n = 4); *p<0.05 compared to control.

^1^O_2_ generated by the photoexcited photosensitizers has a short lifespan and therefore preferentially damages structures in close proximity to the photosensitizer; thus differences between the more hydrophilic ZnTM-2-PyP and the amphiphilic ZnHex-2-PyP in suppression of respiration may arise from different localization of the photosensitizers. One way to test such a possibility is to study how Zn porphyrin analogs affect specific mitochondrial complexes. Cytochrome *c* oxidase (complex IV) is the terminal enzyme in the respiratory electron transport chain in mitochondria and is a potential target for photodynamic therapy [30]. Its photoinactivation would imply that the photosensitizer is localized at a close proximity to the complex. **Fig. 5** shows that illumination of intact cells preincubated with 5 µM ZnTnHex-2-PyP, caused ∼ 50% loss of cytochrome c oxidase activity. At the same concentration, ZnTM-2-PyP was ineffective. ZnTnBu-2-PyP which is ∼ three orders of magnitude more lipophilic than the methyl analog (**Table 1**), demonstrated intermediate efficiency in photoinactivation of cytochrome c oxidase (**Fig. 5**).

**Figure 6:**
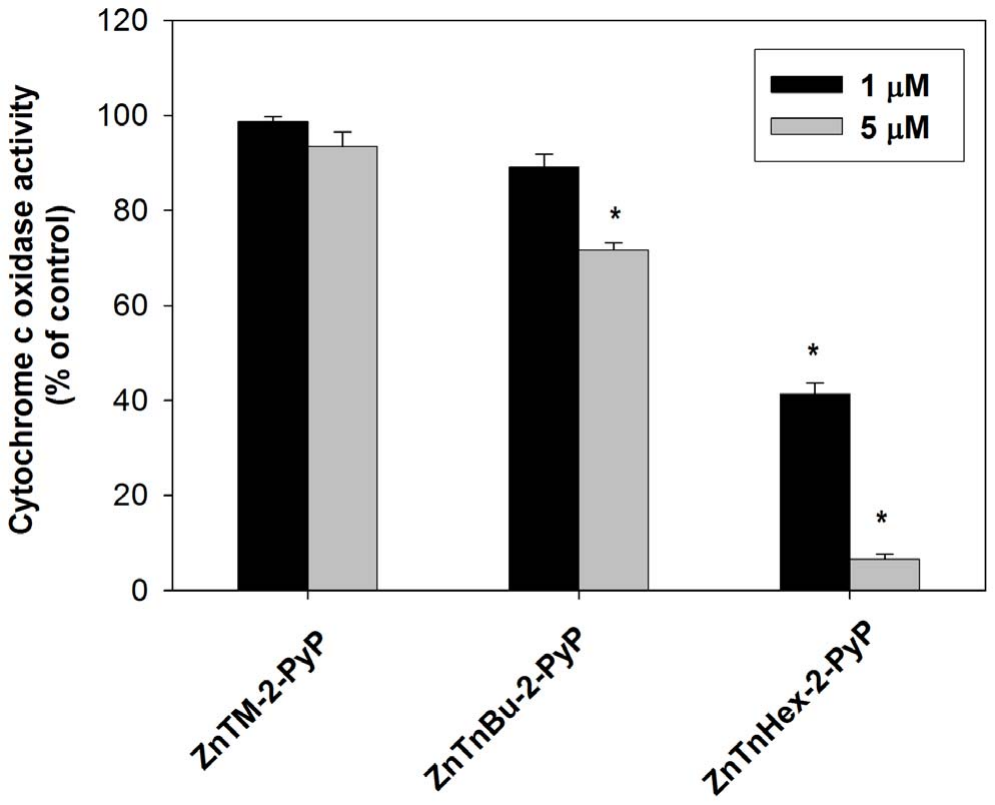
Photoinactivation of cytochrome c oxidase in intact mitochondria. Mitochondria were incubated with 1 and 5 µM ZnTM-2-PyP, ZnTnBu-2-PyP or ZnTnHex-2-PyP for 15 minutes and were then illuminated for 30 minutes. Data are expressed as mean ± SE (n = 4). *p<0.05 compared to control.

Because the cells were washed after preincubation with photosensitizers, the effect on cytochrome c oxidase can be attributed only to the action of Zn porphyrin molecules that had been taken up and remained inside the cells after washing. Intracellular photosensitizer, however, would be shared among various cellular structures with high binding affinity, capable of competing with cytochrome c oxidase. To investigate what fraction of the cell-bound photosensitizer is localized at the vicinity of cytochrome c oxidase, the experiment was repeated with isolated intact mitochondria. In isolated mitochondria, ZnTnHex-2-PyP at 1 µM produced cytochrome c oxidase inactivation comparable to the inactivation caused by 5–10 µM of the same photosensitizer in intact cells (**Fig. 6**). When the same experiment was performed with the hydrophilic methyl analog, no cytochrome c oxidase inactivation was observed even after intact mitochondria were preincubated with 5 µM ZnTM-2-PyP for two hours and then illuminated (not shown). Since at 5 µM ZnTM-2-PyP suppressed oxygen consumption of isolated mitochondria by ∼ 40%, this result suggests that the methyl analog localizes to structures that are involved in electron transport, but not at the cytochrome c oxidase complex.

**Figure 7:**
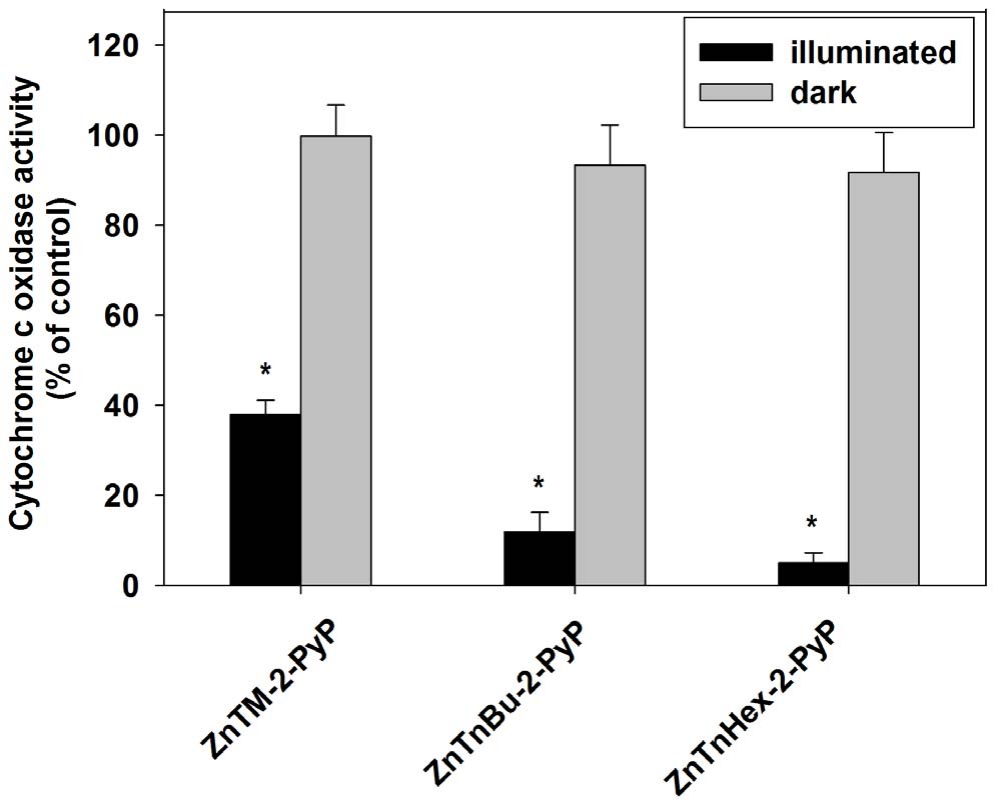
Photo-inactivation of cytochrome c oxidase in permeabilized mitochondria incubated with 10 µM ZnTM-2-PyP, ZnTnBu-2-PyP or ZnTnHex-2-PyP for 30 minutes and then illuminated for 30 minutes. Data are expressed as mean ± SE (n = 4). * p<0.05 compared to control.

To confirm that the observed lack of effect is not due to restricted mitochondrial uptake of the methyl analog, isolated mitochondria were first permeabilized and then incubated with photosensitizers. Even in permeabilized mitochondria, photoinactivation of cytochrome c oxidase was negligible when ZnTM-2-PyP was applied at concentrations up to 5 µM (not shown). When the concentration of the photosensitizers was increased to 10 µM, ∼ 60% of the cytochrome c oxidase activity was lost during 30 min of illumination (**Fig.** **7**). A plausible explanation is that structures distant from cytochrome c oxidase have higher affinity for ZnTM-2-PyP and that after such binding sites are saturated, the excess photosensitizer builds up sufficient concentration in areas where singlet oxygen can reach and inactivate cytochrome c oxidase. In all experiments, no inactivation of cytochrome c oxidase was found when samples were incubated with Zn porphyrins in the dark or were illuminated in the absence of porphyrin.

These results suggest that lipophilicity and overall molecule structure influence not only the uptake, but also the distribution of the photosensitizer to different cellular organelles and molecular complexes. Our findings also indicate that different cellular components vary substantially with respect to affinity for binding exogenous molecules carrying the same overall charge, but having different structural characteristics.

### Specificity of cytochrome c oxidase photoinactivation

It can be speculated that the amphiphilic photosensitizer is taken up very efficiently by mitochondria and thus builds up such a high mitochondrial concentration that it saturates the entire organelle. As a consequence of such abundance of the photosensitizer, illumination causes indiscriminate damage of all components of the mitochondrial membranes and matrix. In this circumstance, photoinactivation of cytochrome c oxidase by ZnTnHex-2-PyP would reflect neither the affinity of the complex nor the distance between the target and the photosensitizer, but is simply a result of the very high concentration of the hexyl derivative in the mitochondria. To test such a possibility, photoinactivation of another mitochondrial marker enzyme, glutamate dehydrogenase, was investigated. Liver glutamate dehydrogenase has been reported to be subject to photoinactivation by methylene blue [31]. Under conditions where cytochrome c oxidase completely lost its activity, none of the Zn porphyrin photosensitizers inactivated glutamate dehydrogenase in native cells, in isolated intact or in permeabilized mitochondria (not shown). This outcome demonstrated that the photoinduced damage of cellular/mitochondrial components is rather selective, with different proteins showing markedly different sensitivities.

## Discussion

Cytochrome *c* oxidase, the terminal complex of the electron transport chain, contains essential histidine residues [32]. Based on the high reactivity of histidine with ^1^O_2_ (*k* = 3.2×10^7^ M^−1^ s^−1^, at pH 7.1) [8], it has been speculated that modification of these residues is the reason for photodynamic inactivation of cytochrome c oxidase. Since photoinactivation of cytochrome c oxidase is caused by photosensitizers with very dissimilar structures, it has been proposed that the inactivation does not require specific binding of the photosensitizer to Complex IV [33]. Such lack of specificity can, however, be attributed to inactivation of cytochrome c oxidase by secondary products of photoinduced lipid peroxidation [34] and reactive products derived from decomposition of protein peroxides [35]. In addition, it has been found that lipophilic cationic porphyrin-based photosensitizers bind directly to cytochrome c oxidase in the mitochondria of intact HeLa cells [36]. Using lifetime resolved fluorescence resonance energy transfer, Börsch demonstrated that PtTM-4-PyP binds directly to cytochrome c oxidase, competing with cyt *c* [36]. PtTM-4-PyP is a platinum *para* analog of ZnTM-2-PyP (**Fig. 1**). We used the *ortho* isomer, ZnTM-2-PyP instead of *para* in our experiments, to avoid distribution of the photosensitizer to cell nucleus [15]. Cellular uptake of the methyl-substituted Zn porphyrin, however, is limited by its hydrophilicity. To improve cellular uptake and to direct the molecule to mitochondria, lipophilicity of the Zn porphyrin was increased ∼five orders of magnitude by replacing the methyl groups with six-carbon aliphatic chains. This modification substantially increased the photodynamic efficiency of the Zn porphyrin. Increased lipophilicity coincided both with more efficient suppression of respiration and with increased inactivation of cytochrome c oxidase. Lipophilic/amphiphilic photosensitizers partition into membranes [5], and upon illumination can inflict photodamage to membrane proteins/protein complexes, including cytochrome c oxidase. Hydrophilic photosensitizers, in contrast, would remain at the water interface, which would hamper their efficiency in damaging membrane components. The more efficient inactivation of cytochrome c oxidase by ZnTnHex-2-PyP compared to ZnTM-2-PyP can therefore be attributed to distribution of the hexyl derivative in the inner mitochondrial membrane. It is noteworthy that another specific mitochondrial protein, the matrix enzyme glutamate dehydrogenase, was not affected by the phototreatment. The lack of effect cannot be due to an intrinsic resistance of glutamate dehydrogenase to photoinactivation since studies with liver glutamate dehydrogenase have demonstrated that the enzyme is readily photoinactivated by methylene blue, as a result of photooxidation of three histidine residues and one tryptophan residue [31]. Like histidine, tryptophan residues react with ^1^O_2_ with a high rate constant (k = 3×10^7^ M^−1^ s^−1^) [8]. The presence of critical amino acid residues which are highly vulnerable to damage by singlet oxygen, and the fact that the enzyme is inactivated by other photosensitizers, strongly supports the idea that the high efficiency of the amphiphilic ZnTnHex-2-PyP in inactivating cytochrome c oxidase results from presence of the photosensitizer in the immediate vicinity of complex IV.

Photoinactivation of cytochrome c oxidase has been shown recently to induce a spurt of mitochondrial superoxide production, which in turn triggers apoptosis in cancer cells [4]. Focused damage to a particular cellular compartment by increased production of reactive species has been shown to avoid systemic side effects, and its advantages for treatment of various diseases have been demonstrated. Therefore, construction of molecules specifically targeting selected macromolecular complexes/organelles and capable of producing bursts of reactive species either by illumination, or via redox cycling [37], has potential for medical application. Improved awareness with respect to cellular sites where such molecules can bind, and an understanding of molecular features that favor distribution and binding to these sites, are prerequisites for rational design of pharmaceutical agents combining high clinical efficiency with minimal side effects.
